# Healthcare Worker Mental Health and Wellbeing During COVID-19: Mid-Pandemic Survey Results

**DOI:** 10.3389/fpsyg.2022.924913

**Published:** 2022-07-14

**Authors:** Kevin P. Young, Diana L. Kolcz, Jennifer Ferrand, David M. O’Sullivan, Kenneth Robinson

**Affiliations:** ^1^Psychological Testing Service, Psychology/Psychiatry, Institute of Living, Hartford Hospital, Hartford, CT, United States; ^2^Wellness, Medical Affairs, Hartford HealthCare, Hartford, CT, United States; ^3^Research Administration, Hartford HealthCare, Hartford, CT, United States; ^4^Emergency Department, Emergency Medicine, Hartford Hospital, Hartford, CT, United States

**Keywords:** healthcare worker (HCW), wellbeing, COVID-19, diversity, depression, anxiety, PTSD

## Abstract

**Introduction/Background:**

HealthCare worker (HCW) mental health and wellbeing are uniquely affected by the complexities of COVID-19 due to exposure to the virus, isolation from family and friends, risk and uncertainty. Little if any inquiry has examined the effects on an entire healthcare system, particularly immediately post-surge. We sought to examine the prevalence of psychiatric symptoms and behavioral health difficulties as a healthcare system transitioned out of the first wave. We assessed the effects of work role, setting and individual diversity factors on employee distress and coping strategies.

**Materials and Methods:**

This was an Institutional Review Board approved, unfunded, voluntary survey sent via REDCap link, to all employees of Hartford HealthCare, a mid-sized healthcare system (*N* ≈ 29,900) between May 15th and June 26th, 2020. Two system-wide emails and two emails targeting managers were sent during this time frame. Eight thousand four hundred and ninety four employees (28.4% of all e-mails distributed) participated in the survey, representing clinical, support, administrative, and medical staff across hospital, outpatient, residential, and business settings. The survey contained items assessing personal background, work environment/culture, and formal measures, including: patient health questionnaire-9 (PHQ-9), general anxiety disorder-7 (GAD-7), primary care post-traumatic stress disorder screen for DSM-5 (PC-PTSD), alcohol use disorders identification test (AUDIT-C), and the insomnia severity index (ISI).

**Results:**

Almost 1/3 of respondents (31%) reported symptoms of clinically significant anxiety; 83% moderate to severe depression; and 51% moderate to severe insomnia. Thirteen percent screened positive for post-traumatic stress disorder. Frontline staff (*p* ≤ 0.001 vs. others) and females (*p* < 0.001 vs. males) endorsed the highest levels of distress, while race (*p* ≤ 0.005) and ethnicity (*p* < 0.03 for anxiety, PTSD and insomnia) had a complex and nuanced interaction with symptoms.

**Conclusion:**

Pandemic stress effects all healthcare employees, though not equally. The effects of work role and environment are intuitive though critical. These data suggest individual diversity factors also play an important role in mental health and wellbeing. All must be considered to optimize employee functioning.

## Introduction

The emotional impact of COVID-19 is widespread and ubiquitous as modern society grapples with a pandemic that has caused devastation unequaled in a century. Early in the pandemic, fear and survival instincts, as mediated by culture and individual factors, drove society’s behavior (Ho et al., 2020).

Concurrently, anxiety and a sense of duty during early days morphed into valiant healthcare worker (HCW) action during the peaks of illness (Ho et al., 2020) all while HCW began accumulating emotional and physical scars (Lai et al., 2020; Lin et al., 2021; Olashore et al., 2021; Young et al., 2021). Initial studies from China (Huang et al., 2020; Lai et al., 2020; Li et al., 2020; Wang et al., 2020), Singapore (Tan et al., 2020), and the United States (Young et al., 2021) detailed high rates of anxiety, depression, trauma-related, and insomnia symptoms among selected segments of society, and HCWs, in particular. One study, conducted in Singapore from February to March, 2020, detailed the emotional effects of COVID-19 on all employees of a health system. Contrary to expectations, non-medical providers had greater emotional symptoms than medical providers, though the sample size was modest and total rates of reported symptoms were significantly less than other available studies of HCW functioning during this time (Tan et al., 2020).

While the virus has continued to overwhelm new areas and additional systems, people of color have been found to be at increased and disproportionate risk of complications from COVID-19 (Centers for Disease Control Prevention, 2020b) sparking dialogue about the factors that cause and maintain healthcare disparities (Centers for Disease Control Prevention, 2020b; Dowling and Kelly, 2020). The impact of these dual threats—a global pandemic and disadvantageous healthcare access—would be expected to increase psychological symptoms for those at higher risk (Wadhera et al., 2020).

This is a unique time in human history and HCWs risk significant emotional sequelae (Lai et al., 2020; Rajabimajd et al., 2021; Young et al., 2021) during a pandemic, because their work responsibilities directly increase their risk of infection and death, when compared to the general population (Centers for Disease Control and Prevention [CDC], 2020a). HCWs who did not receive adequate care subsequent to heightened emotional symptoms during severe acute respiratory syndrome coronavirus 1 (SARS1) continued to suffer at high rates 4 years later (Wu et al., 2009). The breadth and severity of emotional symptoms experienced by HCWs during the sub-acute period (i.e., as a virus wave passes) is not well-understood, though the moment of transition from frantic energy to normalcy may be the key moment for intervention, as anxiety and action presumably shift toward either recovery and resilience or trauma and depression. While stressors would be expected to have an additive effect on mental health, it is unclear whether mental health outcomes are impacted by factors such as job role/exposure, ethnicity, and/or gender.

Our study had several aims. First, we sought to examine the prevalence of psychiatric symptoms as a healthcare system transitioned out of the first wave of the COVID-19 pandemic, but remained uncertain about the future. We hypothesized that depression would be greater, and anxiety diminished, as compared to published data on mid-surge functioning, as at this timepoint, acute stressors were lessened but the psychological impact remained (Young et al., 2021). As the pandemic continued, we sought to explore whether diversity factors impacted psychological functioning, with the hypothesis that people of color would report greater psychological symptoms than White colleagues due to increased rate of COVID-19-related complications in this population. Finally, we hypothesized that contrary to findings from Tan et al. (2020), medical providers with direct exposure to COVID-19 patients would report a higher severity of psychiatric symptoms than employees without patient contact, though certain populations, such as Emergency Department (ED) staff, would report lower levels of symptoms (Young et al., 2021).

## Materials and Methods

A 156-item REDCap survey, approved by the Hartford HealthCare Institutional Review Board (HHC-2020-0069) was sent to all hospital employees and was open from May 15th, 2020 until June 26th, 2020. The study was unfunded, but executive leadership sponsored the research and provided support in dissemination and recruitment via two system-wide emails linking to the study and two emails to managers providing details.

The survey’s content included questions concerning demographic characteristics, psychiatric/medical history, COVID-19 exposure, workplace culture/environment, and formal measures, including the Patient Health Questionnaire-9 (PHQ-9) (Kronkey et al., 2001), General Anxiety Disorder-7 (GAD-7) (Spitzer et al., 2006), primary care post-traumatic stress disorder screen for DSM-5 (PC-PTSD) (Prins et al., 2015), alcohol use disorders identification test (AUDIT-C) (Centers for Disease Control and Prevention [CDC], 2020a), and the insomnia severity index (ISI) (Morin et al., 2011).

### Inclusion Criteria/Participation

All employees (n≈29,900) were invited to participate in the study. A total of 65,685 emails were sent during the recruitment period, including initial emails to all staff, follow-up emails to staff members who did not open the first email, and two emails to all managerial staff. Thirty five thousand six hundred and sixty five (54.3%) of the recruitment emails were opened, and the link to the consent form was clicked 9,088 times. Of the 9,088 who linked to the consent form, 8,494 individuals (93.5%) consented to participate, for an overall response rate of 28.4%.

### Statistical Analysis

REDCap data were exported to and analyzed with SPSS v. 26 (IBM; Armonk, NY 2019). Categorical comparisons were evaluated with a chi square test. Continuous data were evaluated for distribution and analyzed with one of the following, depending on number of groups and normality of distribution: Student’s *t*-test or Mann–Whitney U test for two groups, and analysis of variance or Kruskal–Wallis H test for >2 groups. Correlations were evaluated with a Spearman rank correlation coefficient. A forward, conditional logistic regression model was constructed to evaluate the strength of contribution of many of the variables with univariate differences on outcomes of at least moderate symptoms of anxiety, depression, insomnia, or PTSD. Odds ratios (OR) and 95% confidence intervals (CI) were calculated. All results yielding *p* < 0.05 were deemed statistically significant. Since the number of responses was not known initially, no *a priori* power analysis was performed.

## Results

Of the total 8,494 participants in our study, 83% identified as female, 86% identified as white, 58% were married, and 16% were at least age 60. Thirty-one percent were nurses, 12% were mental health providers, 7% were physicians, and 10% worked in administration. Thirteen percent worked on medical floors, 7% in the ED, and 5% in the ICU. Complete demographic data are shown in Table 1.

**TABLE 1:** Demographic data of respondents.

Characteristic	*N*	Valid%
**Race**		
American Indian/Alaska Native	31	0.5
Asian	176	2.6
Native Hawaiian/Other Pacific Islander	11	0.2
Black/African American	515	7.5
White	5,885	85.7
More than one race	252	3.7
Total	6,870	
Unknown/not reported	291	
**Ethnicity**		
Hispanic or Latino	728	10.5
Not Hispanic of Latino	6,229	89.5
Total	6,957	
Unknown/not reported	1,532	
**Gender**		
Female	6,045	83.4
Male	1,199	16.6
Total	7,259	
Missing	1,230	
**Job type**		
Physician or APP	520	7.2
Nursing staff	2,254	31.4
Clinical staff	1,736	24.1
Support staff	1,915	26.6
Administration	721	10.0
Trainee/Resident/Fellow	43	0.6
Total	7,189	
Missing	1,300	
**Work location**		
Hospital ED	509	7.1
Hospital ICU	374	5.2
Hospital medical floor	907	12.6
Hospital other	2,028	28.1
Office	1,084	15.0
Other	788	10.9
Senior living	87	1.2
Urgent care/Walk-in	37	0.5
Outpatient medical setting	909	12.6
Both inpatient and outpatient	490	6.8
Total	7,213	
Missing	1,276	

### Overview of Emotional Functioning

Thirty-one percent of participants (*n* = 2,023) reported moderate to severe symptoms of anxiety (≥10 on the GAD-7), while 29% (*n* = 1,888) reported mild symptoms (GAD-7 score 5–9). Of note, 23% of the survey participants did not respond.

Eighty-three percent of participants (*n* = 5,285) endorsed moderate to severe depressive symptoms (≥10 on the PHQ-9), although 28% did not respond. Seventeen percent (*n* = 1,046) endorsed mild depression (PHQ-9 score 5–9). Regarding the PHQ-9 question regarding frequency of suicidal ideation, 4.5% (*n* = 279) of respondents answered “several days” or more.

About one eighth (*n* = 820; 13%) of valid surveys contained positive responses to the PC-PTSD scale (score of ≥4), although 25% did not respond. Half of participants (*n* = 2,749; 51%) reported moderate to severe insomnia (scored at least 15 on the ISI). However, 36% of the survey participants did not respond.

While only 54% of study participants responded to questions regarding alcohol use, of those who responded, 49% (*n* = 2,220) endorsed heavy drinking over the past year (scored ≥ 3F; ≥4M on the AUDIT-C).

### Symptom Severity by Demographic and Occupational Characteristics

Females reported experiencing more severe levels of anxiety (33 vs. 22% reported moderate to severe symptoms; *p* < 0.001), depression (85 vs. 74%; *p* < 0.001), PTSD (13 vs. 11%; *p* = 0.023), and insomnia (52 vs. 46%; *p* < 0.001) than men (Table 2). Individuals who identified as Hispanic reported greater anxiety (38 vs. 30% reported moderate to severe symptoms; *p* < 0.001), PTSD (16 vs. 13%; *p* = 0.01), and insomnia (54 vs. 51%; *p* = 0.026) than non-Hispanic individuals. African American individuals endorsed high levels of anxiety (36%) and insomnia (50%), while those who identified as White endorsed high levels of depression (84%; *p* < 0.001).

**TABLE 2:** Rates of depression, anxiety, post-traumatic stress disorder (PTSD), and insomnia by demographics.

Severity category	Total No. (%)	Gender			Ethnicity			Race						
		No. (%)			No. (%)			No. (%)						
		Male	Female	*P*	Hispanic	Non-Hispanic	P	Black	Asian	White	American Indian/Alaska native	Native Hawaiian/Other Pacific Islander	Multiracial	*P*
**GAD-7**														
Minimal	2,582 (40%)	567 (52%)	2,005 (37%)	0.001	219 (37%)	2,265 (40%)	0.001	173 (41%)	69 (45%)	2,129 (40%)	13 (54%)	3 (27%)	71 (33%)	0.005
Mild	1,888 (29%)	284 (26%)	1,596 (30%)		155 (26%)	1,664 (30%)		97 (23%)	46 (30%)	1,586 (30%)	5 (21%)	3 (27%)	56 (26%)	
Moderate	1,096 (17%)	150 (14%)	941 (18%)		100 (17%)	957 (17%)		70 (17%)	18 (12%)	913 (17%)	3 (13%)	3 (27%)	49 (23%)	
Severe	927 (14%)	95 (9%)	828 (15%)		124 (21%)	748 (13%)		79 (19%)	21 (14%)	710 (13%)	3 (13%)	2 (18%)	41 (19%)	
**PHQ-9**														
Minimal	29 (1%)	17 (2%)	12 (0.2%)	0.001	0 (0%)	28 (1%)	0.166	1 (0.3%)	2 (1%)	25 (1%)	0 (0%)	0 (0%)	0 (0%)	0.001
Mild	1,046 (17%)	271 (26%)	770 (15%)		98 (18%)	900 (17%)		96 (25%)	32 (23%)	820 (16%)	4 (19%)	1 (9%)	36 (18%)	
Moderate	2,610 (43%)	437 (41%)	2,165 (43%)		213 (39%)	2,311 (43%)		148 (39%)	60 (43%)	2,216 (44%)	9 (43%)	3 (27%)	67 (34%)	
Moderate/Severe	1,479 (24%)	206 (20%)	1,269 (25%)		143 (26%)	1,279 (24%)		75 (20%)	23 (16%)	1,250 (25%)	6 (29%)	4 (36%)	53 (27%)	
Severe	947 (16%)	128 (12%)	811 (16%)		87 (16%)	817 (15%)		62 (16%)	23 (16%)	759 (15%)	2 (10%)	3 (27%)	43 (22%)	
**PC-PTSD**														
Positive	825 (13%)	118 (11%)	702 (13%)	0.023	95 (16%)	687 (13%)		66 (16%)	20 (14%)	638 (12%)	5 (22%)	2 (18%)	41 (20%)	0.005
Negative	5,533 (87%)	970 (89%)	4,541 (87%)		492 (84%)	4,831 (88%)	0.010	344 (84%)	126 (86%)	4,603 (88%)	18 (78%)	9 (82%)	167 (80%)	
**ISI**														
Absence	340 (6%)	86 (9%)	253 (6%)	0.001	35 (8%)	284 (6%)	0.026	34 (11%)	16 (13%)	259 (6%)	2 (10%)	1 (14%)	9 (5%)	0.001
Subthreshold	2,315 (43%)	429 (45%)	1,876 (42%)		176 (38%)	2,051 (43%)		119 (39%)	51 (43%)	1,978 (44%)	10 (48%)	3 (43%)	59 (36%)	
Moderate	1,946 (36%)	321 (34%)	1,620 (37%)		163 (35%)	1,716 (36%)		100 (33%)	43 (36%)	1,644 (36%)	8 (38%)	2 (29%)	55 (33%)	
Severe	803 (15%)	111 (12%)	682 (15%)		86 (19%)	688 (15%)		52 (17%)	10 (8%)	647 (14%)	1 (5%)	1 (14%)	43 (26%)	
**AUDIT-C**														
Normal	2,321 (51%)	451 (57%)	1,870 (50%)	0.001	197 (53%)	2,044 (51%)	0.300	147 (62%)	42 (64%)	1,939 (50%)	10 (63%)	2 (33%)	74 (58%)	0.001
Severe	2,220 (49%)	337 (43%)	1,883 (50%)		172 (47%)	1,998 (49%)		90 (38%)	24 (36%)	1,960 (50%)	6 (38%)	4 (67%)	54 (42%)	

Individuals working in the ICU and on medical floors endorsed the highest severity of depression, anxiety, PTSD, and insomnia; ED, office workers, and those working in outpatient settings endorsed the lowest severity (Table 3). Nurses and clinical staff endorsed the greatest symptoms of depression and anxiety, while nurses also endorsed high levels of PTSD and insomnia.

**TABLE 3:** Rates of depression, anxiety, post-traumatic stress disorder (PTSD), and insomnia by job type.

Severity category	Total No. (%)	Work location							Job type					
		No. (%)							No. (%)					
		ED	ICU	Medical floor	Outpatient setting	Senior living	Both inpatient and outpatient	*P*	Physician or APP	Nurse staff	Clinical staff	Support staff	Admin	*P*
**GAD-7**														
Minimal	2,582 (40%)	189 (41%)	108 (32%)	257 (32%)	571 (40%)	27 (34%)	199 (45%)	0.001	233 (49%)	719 (36%)	587 (38%)	686 (41%)	309 (47%)	0.001
Mild	1,888 (29%)	136 (30%)	96 (29%)	245 (31%)	408 (28%)	21 (27%)	121 (27%)		144 (30%)	600 (30%)	422 (27%)	500 (30%)	190 (29%)	
Moderate	1,096 (17%)	72 (16%)	65 (19%)	154 (19%)	249 (17%)	13 (17%)	68 (15%)		66 (14%)	359 (18%)	284 (18%)	272 (16%)	99 (15%)	
Severe	927 (14%)	60 (13%)	66 (20%)	142 (18%)	218 (15%)	18 (23%)	53 (12%)		37 (8%)	332 (17%)	265 (17%)	223 (13%)	58 (9%)	
**PHQ-9**														
Minimal	29 (1%)	1 (0.2%)	0 (0%)	2 (0.3%)	6 (0.4%)	0 (0%)	4 (1%)	0.003	6 (1%)	0 (0%)	2 (0.1%)	1 (0.1%)	20 (3%)	.001
Mild	1,046 (17%)	80 (19%)	35 (12%)	88 (12%)	236 (17%)	16 (23%)	85 (21%)		82 (18%)	244 (13%)	233 (16%)	331 (21%)	132 (21%)	
Moderate	2,610 (43%)	187 (44%)	131 (43%)	305 (41%)	584 (43%)	26 (37%)	179 (43%)		228 (49%)	790 (42%)	654 (45%)	610 (39%)	283 (44%)	
Moderate/Severe	1,479 (24%)	101 (24%)	77 (25%)	202 (27%)	325 (24%)	17 (24%)	91 (22%)		93 (20%)	513 (28%)	327 (22%)	385 (24%)	142 (22%)	
Severe	947 (16%)	61 (14%)	62 (20%)	140 (19%)	221 (16%)	12 (17%)	54 (13%)		58 (12%)	316 (17%)	248 (17%)	251 (16%)	60 (9%)	
**PC-PTSD**														
Positive	825 (13%)	63 (14%)	73 (22%)	130 (17%)	181 (13%)	12 (16%)	52 (12%)	0.001	40 (9%)	333 (17%)	198 (13%)	191 (12%)	52 (8%)	0.001
Negative	5,533 (87%)	383 (86%)	258 (78%)	647 (83%)	1,240 (87%)	65 (84%)	375 (88%)		432 (92%)	1,629 (83%)	1,323 (87%)	1,457 (88%)	597 (92%)	
**ISI**														
Absence	340 (6%)	23 (6%)	14 (5%)	29 (5%)	95 (8%)	5 (8%)	24 (7%)	0.002	44 (11%)	74 (5%)	80 (6%)	101 (7%)	36 (6%)	0.001
Subthreshold	2,315 (43%)	156 (41%)	106 (37%)	246 (38%)	516 (42%)	28 (45%)	177 (49%)		195 (47%)	635 (39%)	573 (44%)	604 (44%)	263 (45%)	
Moderate	1,946 (36%)	127 (34%)	106 (37%)	247 (40%)	437 (36%)	21 (34%)	112 (31%)		143 (34%)	643 (39%)	439 (34%)	475 (34%)	222 (38%)	
Severe	803 (15%)	71 (19%)	60 (21%)	115 (18%)	175 (14%)	8 (13%)	46 (13%)		37 (9%)	286 (18%)	204 (16%)	200 (15%)	62 (11%)	
**AUDIT-C**														
Normal	2,321 (51%)	162 (49%)	109 (44%)	296 (52%)	519 (50%)	24 (59%)	160 (50%)	0.355	195 (52%)	694 (49%)	578 (54%)	582 (53%)	246 (48%)	0.033
Severe	2,220 (49%)	166 (51%)	138 (56%)	274 (48%)	513 (50%)	17 (42%)	160 (50%)		177 (48%)	734 (51%)	497 (46%)	517 (47%)	266 (52%)	

### Risk Factors of Mental Health Outcomes

Variables that were associated with our primary outcomes (e.g., depression, anxiety, PTSD, insomnia) *via* univariate analysis were entered into logistic regression models to evaluate the relative contribution of each factor to each outcome. Categorical variables were either dichotomous (age ≥ 60, gender, mental health professional, emergency medicine professional, presence of CDC-defined medical risk factors at time of survey, endorsement of psychiatric history, feeling supported at work, endorsing barriers to working, quarantine of at least a week, adequate access to personal protective equipment (PPE), and feeling the ability to say no to uncomfortable work demands) or multi-level and compared to a reference group. Significant contributors to each model are in Table 4.

**TABLE 4:** Logistic regressions for depression, anxiety, post-traumatic stress disorder (PTSD), and insomnia.

Variable	β	SE	Wald	*p*	OR	95% CI
**Depression**						
Age ≥ 60	–0.325	0.108	9.105	0.003	0.72	0.59–0.89
Female	0.526	0.103	26.223	< 0.001	1.69	1.38–2.07
Mental health professional	–0.318	0.158	4.076	0.044	0.73	0.53–0.99
Prior mental health history	1.091	0.112	94.564	< 0.001	2.98	2.39–3.71
Perceived risk of getting COVID-19			13.475	0.001		
Moderate	0.313	0.101	9.676	0.002	1.37	1.12–1.67
High	0.386	0.123	9.893	0.002	1.47	1.16–1.87
Endorsed barriers to working	0.759	0.091	69.473	< 0.001	2.14	1.79–2.55
Away from home for at least 1 week	0.332	0.105	10.120	0.001	1.39	1.14–1.71
Have access to adequate PPE	–0.322	0.097	10.974	0.001	0.73	0.60–0.88
Can say no to work demands	–0.645	0.091	49.754	< 0.001	0.53	0.44–0.63
**Anxiety**						
Age ≥ 60	–0.622	0.116	29.030	< 0.001	0.54	0.43–0.67
Female	0.280	0.102	7.503	0.006	1.32	1.08–1.62
Prior mental health history	1.020	0.072	200.668	< 0.001	2.77	2.41–3.19
Perceived risk of getting COVID-19			53.913	< 0.001		
Moderate	0.402	0.093	18.925	< 0.001	1.50	1.25–1.79
High	0.751	0.102	53.801	< 0.001	2.12	1.73–2.59
Perceived risk of having COVID-19 complications			8.491	0.014		
Moderate	0.117	0.083	1.998	0.158	1.12	0.96–1.32
High	0.316	0.110	8.172	0.004	1.37	1.10–1.70
Have emotional support	–0.510	0.138	13.58	< 0.001	0.60	0.46–0.79
Endorsed barriers to working	0.771	0.076	102.69	< 0.001	2.16	1.86–2.51
Away from home for at least 1 week	0.266	0.075	12.73	< 0.001	1.31	1.13–1.51
Have access to adequate PPE	–0.366	0.073	25.356	< 0.001	0.69	0.60–0.80
Can say no to work demands	–0.493	0.076	41.848	< 0.001	0.61	0.53–0.71
**PTSD**						
Age ≥ 60	–0.523	00.158	11.016	0.001	0.59	0.44–0.81
Prior mental health history	0.689	0.094	54.008	< 0.001	1.99	1.66–2.39
Perceived risk of getting COVID-19			37.791	< 0.001		
Moderate	0.236	0.132	3.183	0.074	1.27	0.98–1.64
High	0.751	0.137	30.093	< 0.001	2.12	1.62–2.77
Perceived risk of having COVID-19 complications			21.109	< 0.001		
Moderate	0.399	0.107	13.98	< 0.001	1.49	1.21–1.84
High	0.517	0.135	14.76	< 0.001	1.68	1.29–2.18
Have emotional support	–0.482	0.163	8.773	0.003	0.62	0.45–0.85
Endorsed barriers to working	0.526	0.103	26.039	< 0.001	1.69	1.38–2.07
Away from home for at least 1 week	0.510	0.096	28.441	< 0.001	1.67	1.38–2.01
Can say no to work demands	–0.437	0.103	17.992	< 0.001	0.65	0.53–0.79
**Insomnia**						
Age ≥ 60	–0.407	0.096	17.859	< 0.001	0.67	0.55–0.80
Female	0.202	0.090	5.031	0.025	1.22	1.03–1.46
Prior mental health history	0.703	0.072	95.287	< 0.001	2.02	1.76–2.33
Perceived risk of getting COVID-19			21.518	< 0.001		
Moderate	0.301	0.082	13.520	< 0.001	1.35	1.15–1.59
High	0.421	0.096	19.183	< 0.001	1.52	1.26–1.84
Perceived risk of having COVID-19 complications			11.159	0.004		
Moderate	0.226	0.080	8.032	0.005	1.25	1.07–1.47
High	0.281	0.113	6.139	0.013	1.324	1.06–1.65
Have emotional support	–0.509	0.147	12.017	0.001	0.60	0.45–0.80
Endorsed barriers to working	0.315	0.069	20.653	< 0.001	1.37	1.20–1.57
Away from home for at least 1 week	0.227	0.075	9.320	0.002	1.26	1.09–1.45
Can say no to work demands	–0.283	0.070	16.488	< 0.001	0.75	0.66–0.86

*Anxiety was defined as GAD-7 ≥ 10 (at least moderate symptoms), depression was defined as PHQ–9 ≥ 10 (at least moderate symptoms), PTSD was defined as PC-PTSD score of ≥ 4, and insomnia was defined as ISI ≥ 16.*

History of mental illness was an important predictor for all four outcomes (all ORs 1.9–3.0), as was endorsement of real-world barriers to working (all ORs 1.4–1.7), such as concerns about childcare (20%), and feeling unable to say no to work demands (*p* < 0.001). Age also was important, with significant influence (*p* < 0.005) in all four outcomes; older people (i.e., those ≥60) had lower levels of all four outcomes compared with their younger (<60) counterparts. Gender played a role in all outcomes except for PTSD. Concerns about risk of becoming infected, or having a complicated illness course if infected, contributed to all models. Access to PPE had a significant role (*p* ≤ 0.001) in depression and anxiety.

### Sources of Emotional Support

When asked where HCWs turn to for emotional support during COVID-19, a majority of respondents reported that they rely on family (68%) and friends (51%), although many also depend on work leadership (22%) and work peers (42%). However, many fewer individuals reported turning to specialized work programs, such as EAP (employee assistance program) or support groups (3.4%), or private mental health providers (5.3%).

Females, non-Hispanic individuals, and individuals who identify as White endorsed the greatest amount of emotional support and were most likely to seek out mental health treatment. Individuals who identified as White also endorsed significantly greater use of specialized work programs (*p* = 0.001). Conversely, individuals who identified as Black (21%; *p* < 0.001) and/or Hispanic (11%; *p* = 0.032) were significantly more likely to turn to religious groups for support (compared to 8% of total sample that identified religious groups as a source of support). See Table 5 for additional information.

**TABLE 5:** Sources of support endorsed by demographics.

Sources of support	Total No. (%)	Gender			Ethnicity			Race						
		No. (%)			No. (%)			No. (%)						
		Male	Female	*P*	Hispanic	Non-Hispanic	*P*	Black	Asian	White	American Indian/Alaska native	Native Hawaiian/Other Pacific Islander	Multiracial	*P*
**Have at least one source of support**	6548 (77%)	1040 (87%)	5479 (91%)	<0.001	606 (83%)	5673 (91%)	<0.001	430 (83%)	158 (90%)	5386 (92%)	24 (77%)	10 (91%)	217 (86%)	<0.001
Family	5,796 (68%)	923 (77%)	4,849 (80%)	0.011	523 (72%)	5,044 (81%)	<0.001	368 (71%)	142 (81%)	4,794 (81%)	20 (65%)	10 (91%)	183 (73%)	<0.001
Friends	4,335 (51%)	608 (51%)	3,705 (61%)	<0.001	312 (43%)	3,873 (62%)	<0.001	256 (50%)	94 (53%)	3,686 (63%)	11 (35%)	7 (64%)	127 (50%)	<0.001
Work leadership	1,864 (22%)	325 (27%)	1,531 (25%)	0.197	142 (20%)	1,671 (27%)	<0.001	96 (19%)	33 (19%)	1,625 (28%)	7 (23%)	5 (45%)	40 (16%)	<0.001
Work peers	3,525 (42%)	490 (41%)	3,018 (50%)	<0.001	264 (36%)	3,152 (51%)	<0.001	165 (32%)	65 (37%)	3,049 (52%)	8 (26%)	6 (55%)	103 (41%)	<0.001
Specialized work programs	291 (3.4%)	44 (4%)	245 (4%)	0.536	25 (3%)	256 (4%)	0.381	12 (2%)	5 (3%)	241 (4%)	0 (0%)	3 (27%)	9 (4%)	0.001
Religious groups	633 (7.5%)	87 (7%)	543 (9%)	0.053	77 (11%)	513 (8%)	0.032	110 (21%)	14 (8%)	423 (7%)	4 (13%)	1 (9%)	41 (16%)	<0.001
Private mental health worker	454 (5.3%)	49 (4%)	403 (7%)	0.001	33 (5%)	400 (6%)	0.046	21 (4%)	3 (2%)	392 (7%)	1 (3%)	1 (9%)	17 (7%)	0.028
Other	189 (2.2%)	45 (4%)	141 (2%)	0.004	20 (3%)	154 (2%)	0.653	21 (4%)	6 (3%)	132 (2%)	3 (10%)	0 (0%)	9 (4%)	0.008

*N answering each item: Gender N = 7,244; Race N = 6,870; Ethnicity N = 6,957; All study participants N = 8,489.*

Different demographic factors were entered into a logistic regression to determine the relative contribution of each factor on receiving/seeking out support. Race had the biggest influence on whether an individual received (or sought) support, followed by gender and ethnicity. See Table 6.

**TABLE 6:** Logistic regressions for support received/sought out.

Variable	β	SE	Wald	*p*	OR	95% CI
Male	–0.460	0.103	19.962	< 0.001	0.63	0.52–0.77
Non-Hispanic	0.600	0.139	18.788	< 0.001	1.82	1.39–2.39
Race (reference: White)			27.647	< 0.001		
Black	–0.683	0.138	24.598	< 0.001	0.51	0.39–0.66
Asian	0.006	0.285	0.000	0.983	1.01	0.58–1.76
American Indian/Alaska Native	–0.718	0.464	2.39	0.122	0.49	0.20–1.21
Native Hawaiian/Other Pacific Islander	0.327	1.056	0.096	0.757	1.39	0.18–10.98
More than one race	–0.298	0.207	2.069	0.150	0.74	0.50–1.11

## Discussion

### Symptom Prevalence

Reports of moderate to severe anxiety symptoms (31%) was consistent with our national data (Young et al., 2021) and with a meta-analysis of surveys of the public across the world (31.9%) (Salari et al., 2020), although much lower than the 45% reported in Lai et al.’s study from China (Lai et al., 2020). Current rates of anxiety were much greater than the prevalence of generalized anxiety outside of the pandemic (3–18%) (Kessler et al., 2005; Harvard Medical School, 2007).

Eighty-three percent of participants in this study reported moderate to severe depressive symptoms, compared to 14% reported in our national survey (Young et al., 2021), 50% in China (Lai et al., 2020), and 33.7% in the world population (Salari et al., 2020). The rates of suicidal ideation (5%) endorsed in this study was consistent with our national data (Young et al., 2021). The degree of insomnia reported by our participants (51% reported moderate to severe symptoms) is significantly greater compared to the prevalence of insomnia outside of the pandemic (approximately 30% have some symptoms) (Bhaskar et al., 2016). Similarly, of our participants who responded to questions about alcohol use, 49% endorsed heavy drinking during this time. This is significantly higher than the 12.7% of United States population who meet criteria for alcohol use disorder prior to the pandemic (Grant et al., 2017). Lastly, the prevalence of PTSD symptoms reported in this study (13%) was similar to our national data (Young et al., 2021) and much lower than the prevalence of 27% in China (Lai et al., 2020), although much greater compared to the 3.5% prevalence of PTSD in the US outside of the pandemic (Kessler et al., 2005).

The differences in the prevalence of psychiatric symptoms between the studies are multifactorial. The pandemic hit China before the US and very little was known about COVID-19 at that time. As time passed, we came to understand virulence, modes of transmission, optimal PPE, etc. By the time of our survey, increased knowledge and guidance might have resulted in the HCWs feeling less anxious.

The higher level of depression in this study may be related to the timing of the survey as compared to prior research. The study in China (Lai et al., 2020) occurred a few months into the pandemic while case volumes were high, the disease poorly characterized, and risks uncertain. We surveyed staff approximately 3 months into the pandemic locally. The infection had spread globally by that time, but our system’s rates were declining. This supports our hypothesis that as a wave passes, HCWs move from action driven by anxiety and work ethic, to emotional processing of what they have endured; processing that allows time for the pain, death, and disillusionment of the just witnessed tragedy to set in. If this were the case, it would not be surprising for depression and PTSD to increase. It is also possible that the duration of the pandemic caused a more significant impact on the HCWs. Lastly, the higher rates of PTSD in China may reflect a re-experiencing/triggering effect for those who suffered through SARS1.

### Impact of Diversity Factors on Psychological Functioning

In our study, Hispanics reported greater anxiety, PTSD and insomnia than non-Hispanic individuals. As compared to those who identified as White, African-Americans endorsed greater levels of anxiety and insomnia. This may be related to the disproportionately increased risk of COVID-19-related complications among these populations, as well as disparities in access to medical or psychiatric care, access to resources and socio-economic factors. It is also likely that pre-existing inequities, including the sociopolitical, racial, and environmental stressors that these individuals are faced with, are intensified during the pandemic, and extra attention must be given to the unique needs of these populations, as it pertains to the mental health consequences of COVID-19.

It is important to note that by happenstance, this survey was conducted at the same time of an increased awareness of racial inequality, injustice and systemic oppression in our country. Moral outrage, fueled by the ease by which society was able to view and absorb the horrific murder of George Floyd, an unarmed black man killed while in police custody, had fueled a global push for justice and equality not seen in half a century. It is possible that these societal factors had differential effects on HCWs in the study. Higher rates of depression among white HCW could be related to guilt/appreciation of these issues at a time of decreased psychological coping related to the pandemic. Recent surveys of the general population indicated that African- Americans are 20% more likely to experience serious mental health problems than the general population (U S Department of Health and Human Services, 2020), and 40% screened positive for depression and/or anxiety shortly after Floyd’s death (U S Department of Health and Human Services, 2020). It may be that HCWs are uniquely sensitive to these issues.

### Frontline vs. Non-frontline Healthcare Worker Outcomes

In our healthcare system, ICU and medical floor staff endorsed significantly higher levels of depression, anxiety, PTSD, and insomnia, compared to office workers and those working in ED’s or outpatient settings. In addition, clinical staff endorsed the greatest symptoms of depression and anxiety, while nurses also endorsed high levels of PTSD and insomnia ICU and inpatient staff had more frequent and prolonged direct contact with COVID-19 patients, resulting in significant changes to protocols, care areas, staff assignments, and workflow. Working on COVID-19 units requires constant vigilance, incessant donning and doffing of PPE, directly witnessing high rates of suffering and death, and associated feelings of futility despite valiant efforts. Workers without first-hand exposure to COVID-19 patients are probably less aware of the tremendously negative impact of COVID-19, and less direct exposure would be expected to decrease rates of PTSD. It is not surprising that inpatient and ICU nurses, who are overwhelmingly female, and have more intense and prolonged exposure to COVID-19 patients, reported higher levels of PTSD and insomnia.

Interestingly, the risk factors for negatively impacted wellbeing were not entirely work-related. Older age (as a dichotomy) afforded some lessening of severity of outcomes. While very few have experienced the stresses caused by the pandemic, it is possible that older people have a greater tolerance for acceptance of difficult situations. Respondents with a prior mental health history fared worse for all four outcomes, suggesting an increased need for support and/or treatment for this group.

It is interesting that the prevalence of psychiatric symptoms among ED workers was similar to that of outpatient and non-clinical staff. Perhaps healthcare providers with an ability to compartmentalize feelings self-select to work in Emergency Medicine. The pace of work in an ED may prevent fully processing emotional responses to tragedy. While the changes to the ED work environment and processes associated with COVID-19 were impactful, these changes may not have been as extreme a departure from the standard environment and operations as that in other healthcare areas. Additionally, patients with suspected COVID-19 infection are quickly triaged to medical floors, which may minimize ED staff exposure. Lastly, it is also possible that the ED staff are were less likely to admit to these symptoms in this survey.

### Why Might Psychiatric Presentation Change Post-surge?

Our data suggest that as the pandemic wears on, HCWs have become more defeated than afraid. When the survey was administered, the first wave of the pandemic had begun to recede, and the healthcare system had well-established procedures and processes to protect the workforce. By this time, HCWs’ anxiety and uncertainty were likely mitigated by increased knowledge and experience of the virus. Nevertheless, chronic physical and emotional stress can cause depressive symptoms. During the chaos of a pandemic, a decrease in HCW engagement in health behaviors would further drive depression.

The pandemic and associated challenges have had a wearing effect on our healthcare workers, and we have not yet replenished spent resources. Our findings support those from Ettman et al. (2020), who found the prevalence of depression in a general population sample in early April to be three times higher than prior to the pandemic; lower income, less financial security, and number of stressors all increased depression (Ettman et al., 2020). Generally, greater access to resources, whether financial or social, is protective against depression.

Our data show that female gender and real-world barriers to working (i.e., child/elder/pet care, responsibilities at second job, risk of infecting self/others) increase the likelihood of mental health symptoms. This is consistent with national and international data suggesting that while men have worse outcomes and higher mortality from COVID-19, women are more likely to bear the brunt of the social and economic consequences of the pandemic. The women’s advocacy group LeanIn.org surveyed 3,000 American adults in May of 2020 about changes to their everyday lives during the pandemic. Full-time working women with children reported almost 20 more hours a week than men on domestic tasks. The disparity is especially acute for women of color and single women, and only 41% of employees surveyed said that their employer has changed policies to allow more flexibility. This, coupled with the estimation that women comprise 70% of healthcare and social sector jobs worldwide (Boniol et al., 2019), suggests that women HCWs face the most significant burdens physically, socially, and economically during the pandemic.

## Implications

Studies conducted at different points in the pandemic uniformly suggest significant suffering among HCWs. Health care systems play an integral role in caring for their workers and creating a culture that supports wellness, compassion and self-care. We agree with Shanafelt’s notion that burnout prevention in healthcare is a shared responsibility between the organization and its members (Shanafelt and Noseworthy, 2017). If engagement (e.g., vigor, dedication and absorption in work) is the antithesis of burnout, then increasing staff engagement should mitigate some of the psychological symptoms that have resulted from COVID-19. Particularly in times of crisis, it is unreasonable to place the burden of responsibility on HCWs to meet their own needs for psychological wellbeing and support. The organization must not neglect the organizational drivers that contribute to burnout, including culture/values, efficiency of practice and work-life integration, and organizational interventions must prioritize these issues.

Consistent with Shanafelt’s organizational recommendations for burnout prevention, an organizational approach to COVID-19 recovery should start with acknowledging and assessing the problem. We have done so not only by surveying our staff, but also by communicating results to and from the highest level of leadership, along with an organizational commitment to respond. The organization must then must develop and implement specific; in our system those interventions include peer support, training managers to identify and respond to distress, and triaging affected individuals to personalized support services including EAP, as well as pro-bono and highly responsive group and individual behavioral health interventions created and implemented by our own staff. These steps align with recommendations that existed pre-pandemic, including the importance of cultivating community, promoting flexibility and work-life balance, and providing resources to promote resilience and self-care (Shanafelt and Noseworthy, 2017). These efforts must all be made in a way that aligns with the organization’s values, culture and mission, or interventions will be ineffective or poorly utilized.

## Limitations/Future Directions

This study’s participants worked in a single state, although the participants had unique job roles across a diverse system. Our response rate was below our goal, but was similar to the response rate of the same staff on a mandatory organization engagement survey administered during this time. We believe that staff were overwhelmed by demands of the work during this stage in the pandemic, and survey completion, either research or required for job, became a non-priority. Despite that, a wide range of diverse staff completed the survey, and we do not believe there is a reason to suspect that this introduced systematic bias. Finally, the he design was cross-sectional and we hope to follow up with these same HCWs to monitor for change(s) and to more definitively understand factors leading to positive/negative outcomes.

Future research should investigate reasons why some employees in need of support do not engage with services (Young et al., 2021). Additionally, it will be critical to design and study tailored interventions that account for personal, job-specific/job-demand, and symptom/functional status. All stakeholders (staff, trainees, administration) should be involved in development, implementation, and evaluation of future wellness programs.

## Data Availability Statement

The original contributions presented in this study are included in the article/supplementary material, further inquiries can be directed to the corresponding author.

## Ethics Statement

The studies involving human participants were reviewed and approved by Hartford HealthCare Institutional Review Board. The patients/participants provided their written informed consent to participate in this study.

## Author Contributions

KY, DK, and DO’S contributed to the methods. DK and DO’S created the tables. All authors provided unique and vital information to this manuscript, collaborated regarding background, design, results, and discussion, and approved the submitted version.

## Conflict of Interest

The authors declare that the research was conducted in the absence of any commercial or financial relationships that could be construed as a potential conflict of interest.

## Publisher’s Note

All claims expressed in this article are solely those of the authors and do not necessarily represent those of their affiliated organizations, or those of the publisher, the editors and the reviewers. Any product that may be evaluated in this article, or claim that may be made by its manufacturer, is not guaranteed or endorsed by the publisher.
